# Prognostic prediction across a gradient of total tumor volume in patients with hepatocellular carcinoma undergoing locoregional therapy

**DOI:** 10.1186/1471-230X-10-146

**Published:** 2010-12-31

**Authors:** Teh I Huo, Chia Y Hsu, Yi H Huang, Chien W Su, Han C Lin, Rheun C Lee, Yi Y Chiou, Jen H Chiang, Pui C Lee, Shou D Lee

**Affiliations:** 1 Institute of Pharmacology, National Yang-Ming University School of Medicine, Taipei, Taiwan; 2 Institute of Clinical Medicine, National Yang-Ming University School of Medicine, Taipei, Taiwan; 3 Faculty of Medicine, National Yang-Ming University School of Medicine, Taipei, Taiwan; 4 Department of Medicine, Taipei Veterans General Hospital, Taipei, Taiwan; 5 Department of Radiology, Taipei Veterans General Hospital, Taipei, Taiwan; 6 Department of Medicine, National Yang-Ming University Hospital, Yilan, Taiwan

## Abstract

**Background:**

The size and number of tumors are important prognostic indicators for hepatocellular carcinoma (HCC). However, it is difficult to assess the prognosis for patients with a variable number and size of tumors. By combining these two factors, we investigated the role and prognostic accuracy of total tumor volume (TTV) for HCC.

**Methods:**

A total of 786 patients undergoing locoregional therapy (transarterial chemoembolization, percutaneous radiofrequency ablation and acetic acid or ethanol injection) for HCC were prospectively evaluated.

**Results:**

The mean and median TTV was 177 cm^3 ^(range, 0.1-3,591 cm^3^) and 21 cm^3^, respectively. Of all, 38%, 29%, 15%, 7% and 11% of patients had TTV of <10 cm^3^, 10-50 cm^3^, 50-200 cm^3^, 200-500 cm^3 ^and >500 cm^3^, respectively. TTV was significantly larger in patients with higher serum α-fetoprotein (AFP) levels or with vascular invasion. The Child-Turcotte-Pugh score, performance status, vascular invasion, AFP level and TTV were significant independent prognostic predictors in the Cox proportional hazards model. After adjustment, patients with TTV 50-200 cm^3 ^(relative risk [RR]: 1.74, p = 0.009), 200-500 cm^3 ^(RR: 2.15, p = 0.006) and >500 cm^3 ^(RR: 3.92, p < 0.001) had a significantly increased mortality risk in comparison to patients with TTV <10 cm^3^.

**Conclusions:**

TTV is a feasible prognostic predictor across a wide gradient and can be used to predict the mortality risk of HCC. Selecting appropriate cutoffs of TTV may help refine the design of cancer staging system and treatment planning. Future clinical trials of HCC may include this parameter for mortality risk stratification.

## Background

Hepatocellular carcinoma (HCC) is one of the most common malignant tumors in the world [1,2]. The prognosis for patients with HCC is often very poor. Among the reported prognostic predictors for HCC, the size and number of tumor nodule, which represent tumor burden, are frequently associated with the aggressiveness of HCC and are of prime importance in determining the clinical outcome of these patients [3-6]. The size and number of tumor or the extent of tumor involvement have also been included into the staging systems for HCC [7]. In addition, the selection of treatment modality for HCC is also highly dependent on the number and size of tumor nodule. For instance, the Milan criteria are widely accepted standards for HCC patients undergoing liver transplantation [8-10], whereas patients undergoing partial hepatectomy usually have a maximal tumor number of two or three. For patients undergoing percutaneous ethanol or acetic acid injection or radiofrequency ablation, the largest size of tumor is usually set at 3 cm and the number of tumor nodule usually does not exceed 3 [11,12]. However, for patients beyond these criteria, the treatment selection may widely vary and the prognostic prediction may become quite difficult. A major reason for this uncertainty and heterogeneity in cancer therapy is that a single determinant representing tumor burden has not yet been specifically defined. For example, the prognosis for a patient with a single 6 cm-sized HCC may not be the same for a patient with 3 nodules with a tumor diameter of 4, 3 and 2 cm for each nodule. As such, it is difficult to assess the prognosis for patients with a variable number and size of tumor nodules, and this difficulty may make the application of the currently used prognostic models for HCC less practical and clinically feasible. The concept of using total tumor volume (TTV) to represent tumor burden has been proposed by independent study groups [13,14]. By combining the factor of size and number of tumor nodule, we aimed to define the tumor burden by using TTV in assessing the long-term outcome of patients with HCC. In this study, we have investigated the feasibility of TTV, its association with other clinical parameters and its predictive accuracy in patients with HCC undergoing locoregional therapy.

## Methods

### Patients and diagnosis

Patients with newly diagnosed HCC in our hospital were evaluated since April 2002. The clinical parameters in these patients were prospectively assessed and recorded. The diagnosis of HCC was histologically verified by needle biopsy, or based on the findings of typical radiological features in at least two image examinations including ultrasound, contrast-enhanced dynamic computed tomography (CT), magnetic resonance imaging and hepatic angiography, or by a single positive imaging technique associated with serum α-fetoprotein (AFP) level >400 ng/mL [15,16]. The underlying etiology of liver disease was attributed to hepatitis B virus (HBV) infection if patients were seropositive for hepatitis B surface antigen (HBsAg, RIA kits, Abbott Laboratories, North Chicago, IL, USA) and attributed to hepatitis C virus (HCV) infection if patients were seropositive for an antibody against HCV by a second-generation enzyme immunoassay (Abbott Laboratories, IL). The performance status was evaluated using the Eastern Cooperative Oncology Group (ECOG) performance scale: 0 (asymptomatic) to 4 (confined to bed). The model for end-stage liver disease (MELD) score and Child-Turcotte-Pugh (CTP) score were used to estimate the severity of liver cirrhosis according to previous reports [17-20]. The MELD score = 9.57 × log_e _(creatinine mg/dL) + 3.78 × log_e _(bilirubin mg/dL) + 11.2 × log_e _(international normalized ratio of prothrombin time) + 6.43. Minimal values were set to 1.0 for calculation purposes. The maximal serum creatinine level considered within the MELD score equation was 4.0 mg/dL. The CTP classification was based on serum levels of albumin and bilirubin, prothrombin time prolongation, and the severity of ascites and encephalopathy. The Cancer of the Liver Italian Program (CLIP) was used as the staging systems for HCC [7]. The presence of ascites or vascular invasion by the tumors, and the size and number of the each tumor nodule were determined and measured by the contrast-enhanced dynamic CT scan. The volume of a tumor nodule was calculated by using the following mathematical equation:

$${\text{Tumor volume (cm}}^{3}\text{)}=\text{4/3}\times \text{3}.\text{14}\times {\left(\text{maximum radius of the tumor nodule in cm}\right)}^{\text{3}}$$

TTV was the sum of the tumor volumes of every nodule:

$${\text{TTV (cm}}^{\text{3}}\text{)}=\text{Tumor volume of }\left(\text{tumor nodule 1}+\text{tumor nodule 2}+\ldots \ldots +\text{tumor nodule N}\right)$$

### Treatment

All patients with a diagnosis of HCC and admitted to our hospital would undergo initial evaluation for the possibility of surgical resection. For patients with unresectable lesions, curative or palliative locoregional therapy including transarterial chemoembolization (TACE), percutaneous acetic or ethanol acid injection (PAI, PEI), or radiofrequency ablation (RFA) was performed depending on the size and number of tumor nodules. The selection of treatment modality and the details of treatment procedure have been described in our previous studies [6,21-23]. Typically, percutaneous ablation therapies were administered to patients with small (<3 cm) tumor nodule and lesions no more than three. TACE was indicated for patients with intermediate-sized tumor or multi-nodular lesions. Malignant ascites, main portal vein thrombosis by tumor invasion, or extrahepatic tumoral spread indicated advanced cancer stage and were contraindications for any forms of locoregional therapy. Patients with clinical signs of viable or recurrent tumors after treatment were re-evaluated and re-treated using the same or different treatment modality. This study has been approved by the Institutional Review Board of our hospital and complies with the standards of Declaration of Helsinki and current ethical guidelines.

### Statistical analysis

The Chi-squared test or Fisher's exact test was used for categorical data, and the Mann-Whitney ranked sum test was used for continuous data. The Kruskal-Wallis test was used for the comparison across three or more groups. Factors that may be associated with the survival including age, gender, etiology of cirrhosis, CTP score, MELD score, alanine aminotransferase (ALT), aspartate aminotransferase (AST), presence of ascites or vascular invasion, serum AFP level, ECOG performance status and TTV, were included in the univariate survival analysis. TTV was further categorized into five groups, <10 cm^3^, 10-50 cm^3^, 50-200 cm^3^, 200-500 cm^3 ^and >500 cm^3^, to determine its prognostic ability for outcome prediction. Survival analysis between different groups of patients was performed by using the Kaplan-Meir method. The significance and relative risk (RR) of the prognostic factors predictive of the survival in HCC patients were determined and adjusted by using the multivariable Cox proportional hazards model. The adjusted relative risks with 95% confidence intervals (CI) were derived to assess the magnitude of the association between various predictors and the risk or mortality. The discriminatory ability of different staging system was examined by using the Cox proportional hazards model, and the consequences of the Cox model were expressed with the Akaike information criterion (AIC), which reveals how the staging systems affected the dependent variable (patient survival) and represents an overall assessment of a certain staging system [24,25]. The lower the AIC, the more explanatory and informative the model is [26]. Statistical significance levels were determined by 2-tailed tests. A p value < 0.05 was considered statistically significant. All statistical analyses were conducted using SPSS for Windows version 14 (SPSS Inc., Chicago, IL, USA) and MedCalc for Windows version 4.2 (MedCalc Software, Mariakerke, Belgium).

## Results

### Patient profiles

A total of 786 HCC patients who underwent locoregional therapy during the period of April 2002 to June 2008 were enrolled and formed the basis of this study. The baseline demographics of the study patients were shown in Table 1. Patients were predominantly elderly (mean age, 67 years) males (73%). Among them, 50% had evidence of chronic HBV infection and 34% had evidence of chronic HCV infection. The baseline MELD score was 9.3 ± 3.1, and 81% of the patients had a well-preserved liver function (CTP class A). Five hundred (64%) patients had a single tumor nodule, and 72% of patients had a maximal tumor size of 5 cm or smaller. The mean and median TTV was 177 cm^3 ^(range, 0.1-3,591 cm^3^) and 21 cm^3^, respectively. Analysis of the distribution of TTV showed that 38%, 29%, 15%, 7% and 11% of patients had a TTV of <10 cm^3^, 10-50 cm^3^, 50-200 cm^3^, 200-500 cm^3 ^and >500 cm^3^, respectively. Of all patients, 60% had undergone TACE, 28% had undergone RFA and 10% had received PAI or PEI as the primary anti-cancer treatment.

**Table 1:** Baseline demographics

Number of patients	786
	
Age (mean ± SD years; range)	67 ± 12 (27-92)
	
Male/female (%)	73/27
	
Underlying liver disease (%)	
HBsAg-positive	393 (50)
HBsAg-negative	
Anti-HCV-positive	265 (34)
Anti-HCV-negative	128 (16)
	
Tumoral characteristics (%)	
Single lesion	500 (64)
Multi-nodular lesions	286 (36)
Maximal tumor diameter	
≤ 5 cm	566 (72)
>5 cm	220 (28)
	
Ascites (%)	
Yes	101 (13)
No	685 (87)
	
Serum AFP level (ng/mL) (%)	
Median (range)	29 (2-10,032,600)
<20	344 (44)
20 - 400	288 (37)
>400	154 (19)
	
Performance status (%)	
ECOG scale 0	590 (75)
ECOG scale 1-3	196 (25)
	
Serum biochemistries (mean ± SD)	
Albumin (g/dL)	3.7 ± 0.6
Bilirubin (mg/dL)	1.1 ± 1.0
Creatinine (mg/dL)	1.3 ± 3.2
Prothrombin time INR	1.1 ± 0.3
ALT (IU/L)	69 ± 68
AST (IU/L)	52 ± 44
	
Child-Pugh-Turcotte class (%)	
A	639 (81)
B	147 (19)
	
MELD score (mean ± SD; range) (%)	9.3 ± 3.1 (6.4-23.9)
<8	336 (43)
8-11	215 (27)
>11	235 (30)
	
Vascular invasion (%)	
No	699 (9)
Yes	87 (91)
	
CLIP staging (%)	
Score 0	266 (34)
Score 1	276 (35)
Score 2	141 (18)
Score 3	66 (8)
Score 4 or 5	37 (5)
	
Total tumor volume (cm^3^) (%)	
Mean ± SD (range)	177 ± 436 (0.1-3,591)
Median	21
<10	297 (38)
10-50	226 (29)
50-200	121 (15)
200-500	57 (7)
>500	85 (11)
	
Treatment (%)	
PAI or PEI	79 (10)
RFA	221 (28)
TACE	471 (60)
More than one methods	15 (2)

### Distribution and association of TTV and number and size of tumor nodule

The size and number of tumor nodule in relation to TTV were evaluated and shown in Table 2. For patients with uni-nodular or multi-nodular lesions, the TTV significantly increased with increasing maximal tumor diameter in the categories of <3 cm, 3-6 cm and >6 cm (p < 0.0001). For the category of patients with maximal tumor diameter <3 cm, the TTV tended to significantly increase with the number of tumor nodules (p < 0.0001). However, there were no significant differences of the TTV among the four groups (one, two, three and four or more nodules) of patients with maximal diameter of 3-6 cm (p = 0.054) and 6 cm (p = 0.177).

**Table 2:** Distribution of the number and size of tumor and total tumor volume

**No. of tumors and patients, and total tumor volume (cm**^**3**^**)**	Maximal tumor diameter (cm)			p
		
	<3	3-6	>6	
One nodule				
No. of patients	238	148	114	
Total tumor volume	5 ± 4	44 ± 29	730 ± 692	<0.001^a^
(median)	(4)	(34)	(524)	
				
Two nodules				
No. of patients	73	45	32	
Total tumor volume	7 ± 5^d^	53 ± 29	720 ± 746	<0.001^b^
(median)	(6)	(51)	(372)	
				
Three nodules				
No. of patients	34	22	10	
Total tumor volume	7 ± 5^e^	45 ± 29	472 ± 429	<0.001^c^
(median)	(6)	(33)	(278)	
				
Four or more nodules				
No. of patients	34	21	15	
Total tumor volume	10 ± 6^f^	59 ± 41	937 ± 719	
(median)	(11)	(66)	(703)	
p	<0.001	0.054	0.177	

Kruskal-Wallis test with post-test: a, b and c: p values all <0.05 for pairwise comparison between the 3 groups; d vs e, d vs f and e vs f: p values all >0.05.

### Association of TTV and tumoral and cirrhosis parameters

**Figure 1:**
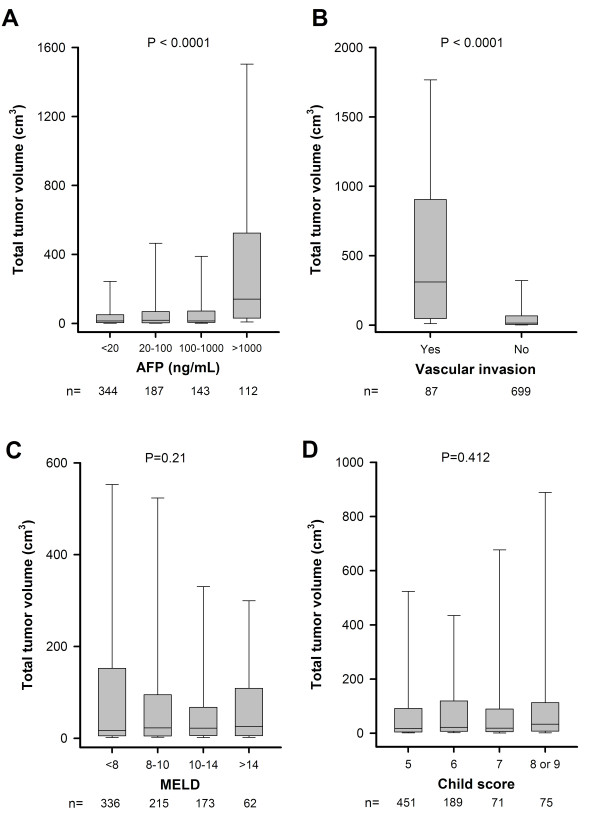
**Association of the total tumor volume (TTV) with serum AFP level (panel A), vascular invasion (panel B), MELD (panel C) and Child score (panel D)**. AFP level and vascular invasion, but not MELD and Child score, were significantly associated with TTV. Data were expressed as median (horizontal bars) and 25% to 75% percentile of the distribution (lower and upper margin of the square); the upper and lower vertical bars indicate 90% and 10% percentile of the distribution, respectively.

To determine the association between TTV and other important prognostic indicators of HCC, the relationship of TTV and serum AFP level, vascular invasion, CTP and MELD score was investigated (Figure 1). TTV significantly increased with increasing serum AFP levels (p < 0.0001). TTV was also significantly higher in patients with vascular invasion (624 ± 774 cm^3^) than patients without vascular invasion (122 ± 334 cm^3^; p < 0.0001). However, there was no significant association between TTV and the MELD (p = 0.21) or CTP (p = 0.412) score. A total of 122 patients had a biopsy to evaluate tumor histology: 39, 69 and 14 patients had well-, moderate- and poorly-differentiated HCC respectively; there was no significant difference among the grade of cell differentiation and TTV (mean values: 89 ± 288, 94 ± 235 and 166 ± 403 cm^3 ^respectively, p = 0.184).

### Survival analysis

**Figure 2:**
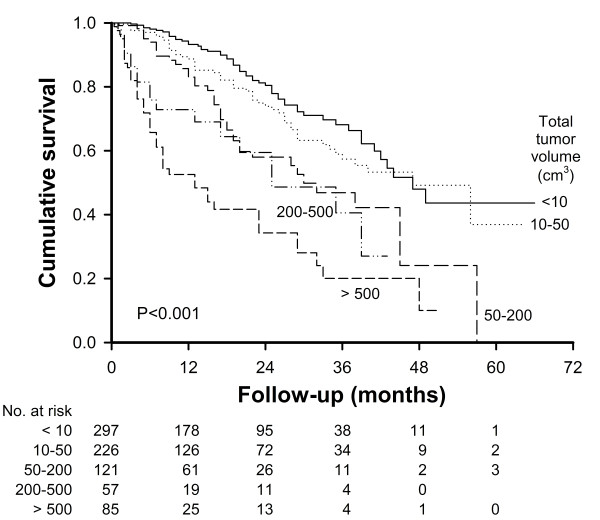
**Comparison of the survival rates between five groups of patients with different total tumor volumes**.

Up until July 2008, 216 (27%) patients died during a mean follow-up period of 18 ± 14 months (range, 1-66 months). In the univariate survival analysis, ascites (p < 0.001), ECOG performance status (p < 0.001), vascular invasion (p < 0.001), AFP level (p < 0.001), MELD score (p = 0.04), CTP score (p < 0.001), AST level (p = 0.001) and TTV (p < 0.001) were significant prognostic predictors (Table 3). In the Cox proportional hazards model, CTP score of 6 or higher (RR: 1.67, p < 0.001), ECOG status 1 or higher (RR: 2.20, p < 0.001), vascular invasion (RR: 2.14, p < 0.001), AFP level >100 ng/mL (RR: 1.50, p = 0.006) were significant independent prognostic factors predicting a poor survival (Table 4). In addition, there was an increasing trend of mortality risk across a gradient of TTV in the Cox model. Compared with patients with TTV <10 cm^3^, the adjusted relative risk for mortality was 1.13 (95% CI: 0.78-1.65, p = 0.514) for patients with TTV 10-50 cm^3^; 1.74 (95% CI: 1.15-2.64, p = 0.009) for patients with TTV 50-200 cm^3^; 2.15 (95% CI: 1.24-3.72, p = 0.006) for patients with 200-500 cm^3^; 3.92 (95% CI: 2.55-6.03, p < 0.001) for patients with TTV >500 cm^3^. The comparison of the survival rates among the five categories of TTV was shown in Figure 2. Patients with the largest TTV had the highest cumulative mortality rate, whereas patients with the smallest TTV tended to have a better survival rate (p < 0.001).

**Table 3:** Significance of the prognostic predictors in the univariate survival analysis

Parameters	No. of patients	Survival rate (%)		p
			
		2-yr	4-yr	
Age (years)				0.795
>60	554	68	39	
≤ 60	232	70	37	
				
Gender				0.713
Male	576	69	42	
Female	210	71	29	
				
HBsAg				0.589
Positive	393	68	42	
Negative	393	70	34	
				
Ascites				<0.001
Yes	101	50	11	
No	685	72	42	
				
Performance status				<0.001
ECOG 0	590	75	45	
ECOG 1 or higher	196	51	10	
				
Vascular invasion				<0.001
Yes	87	35	22	
No	699	72	41	
				
AFP (ng/mL)				<0.001
>100	253	58	33	
<100	533	74	42	
				
MELD score				0.04
>10	234	62	36	
<10	552	72	40	
				
CTP score				<0.001
5	451	75	50	
6 or higher	335	60	25	
				
ALT (IU/L)				0.934
>40	499	70	38	
≤ 40	287	67	40	
				
AST (IU/L)				0.001
>40	551	65	31	
≤ 40	235	77	52	
				
Total tumor volume (cm^3^)				<0.001
<10	297	80	48	
10-50	226	74	47	
50-200	121	56	25	
200-500	57	54	-	
>500	85	34	10	

**Table 4:** Adjusted relative risks of the prognostic predictors in the Cox proportional hazards model

Predictors	Regression coefficient	Standard error	Relative risk	95% CI	p
Child score ≥ 6	0.51	0.15	1.67	1.25-2.23	<0.001
					
ECOG scale ≥ 1	0.79	0.15	2.20	1.63-2.97	<0.001
					
Vascular invasion	0.76	0.20	2.14	1.44-3.19	<0.001
					
AFP > 100 ng/mL	0.40	0.15	1.50	1.13-1.99	0.006
					
Total tumor volume					
<10 cm^3^	-	-	1	-	-
10-50 cm^3^	0.13	0.19	1.13	0.78-1.65	0.514
50-200 cm^3^	0.55	0.21	1.74	1.15-2.64	0.009
200-500 cm^3^	0.77	0.28	2.15	1.24-3.72	0.006
>500 cm^3^	1.37	0.22	3.92	2.55-6.03	<0.001

### Application of TTV in the staging system

**Figure 3:**
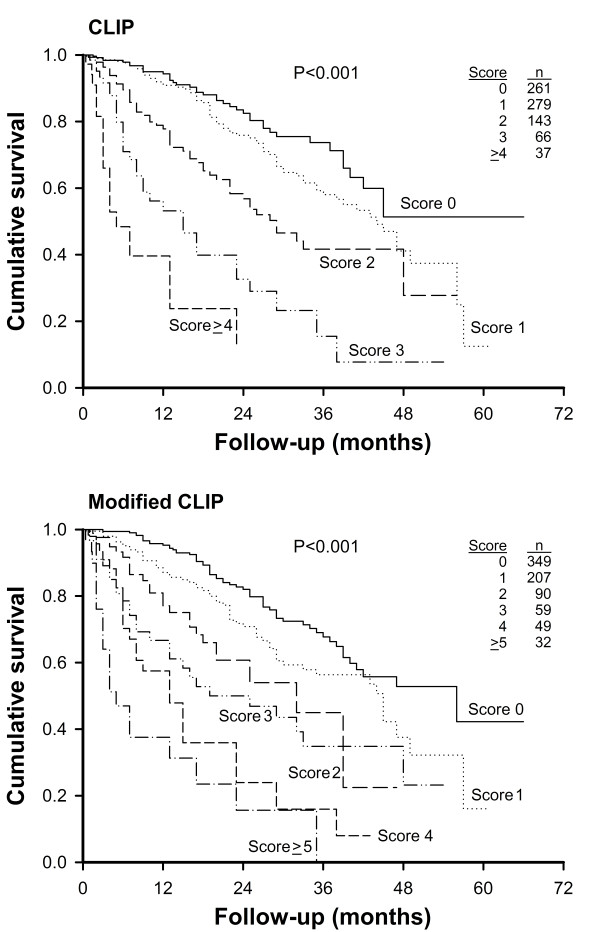
**Comparison of the survival distributions between different score groups in the original and modified CLIP systems**. There was a significant difference in the survival distribution across all score groups in the original (p < 0.001; panel A) and TTV-based (p < 0.001; panel B) CLIP scoring system. The survival significantly tended to be worse with increasing scores in both models.

To further investigate the feasibility of TTV in the cancer staging system, we have replaced the factor of tumor character in the CLIP scoring system with TTV (<50 cm^3^, 50-200 cm^3^, 200-500 cm^3 ^and >500 cm^3^; Table 5). The modified CLIP system used a TTV-based scoring method to re-stage all study patients. The mean scores in the original and modified CLIP system were 1.2 ± 1.2 (range, 0-5) and 1.2 ± 1.5 (range, 0-7), respectively. In the survival analysis, there was a significant difference in the survival distribution across different score groups in the original (p < 0.001; Figure 3A) and TTV-based (p < 0.001; Figure 3B) CLIP scoring system. The survival significantly tended to be worse with increasing scores in both models. The modified CLIP system had a lower AIC value (3875.7) in comparison with the original CLIP model (3969.6), suggesting a better predictive accuracy for the TTV-based model.

**Table 5:** Risk score assessment according to the original and total tumor volume (TTV)-based CLIP scoring system

Parameters	Original CLIP	TTV-based CLIP
Tumor morphology		
Single and <50% liver span	0	-
Multiple and <50% liver span	1	-
≥ 50% liver span	2	-
		
Total tumor volume		
<50 cm^3^	-	0
50-200 cm^3^	-	1
200-500 cm^3^	-	2
>500 cm^3^	-	3
		
Serum AFP level (ng/mL)		
<400	0	0
≥ 400	1	1
		
Macroscopic vascular invasion		
No	0	0
Yes	1	1
		
CTP class		
A	0	0
B	1	1
C	2	2

The score range is 0-6 for the original CLIP and 0-7 for TTV-based CLIP scoring system.

## Discussion

In this study, we have proposed a new prognostic predictor, the TTV, for HCC, and have evaluated its feasibility for outcome prediction. In most published literatures, the traditional way to report the tumor burden of HCC is to describe the size and number of tumor nodule simultaneously. However, it could be quite difficult or even confusing to assess the prognosis of patients with a wide range of number and size of tumor nodules. To solve this problem, the factors of number and size of tumor nodule are transformed into one single parameter in this study. There are several clear advantages of this strategy. Firstly, the TTV represents true tumor burden and can simplify the way to describe the extent of tumor involvement in liver. Secondly, TTV is a continuous variable that can be easily calculated and used to predict the survival more specifically. Alternatively, it can also be categorized into different risk groups in terms of outcome prediction. Thirdly, TTV can be incorporated into the staging system for HCC because there was a significant dose-response relationship between the TTV and long-term mortality risk in our study. Notably, patients with TTV 50-200 cm^3^, 200-500 cm^3 ^and >500 cm^3 ^had a significantly increased mortality risk of 74%, 115% and 292%, respectively, in comparison to patients with TTV <10 cm^3 ^after adjusting for other significant prognostic variables in the Cox analysis. These results indicate that TTV can serve as an independent prognostic predictor for HCC.

In the five risk-stratified TTV groups, we found that patients with TTV <50 cm^3 ^had a lower mortality risk and patients with TTV >200 cm^3 ^had an increased mortality risk; the risk for patients with TTV between 50 to 200 cm^3 ^was considered intermediate. A TTV of 50 cm^3 ^is equivalent to a single tumor nodule with a diameter of 4.6 cm. This cutoff is considered appropriate for clinical application because TTV <50 cm^3 ^is within the Milan criteria, suggesting the risk of tumor recurrence is very low after liver transplantation or partial hepatectomy [9,27]. On the other hand, a TTV of 200 cm^3 ^is equivalent to a single tumor nodule with a diameter of 7.3 cm or three nodules the diameter in each of which being 5 cm. Patients with TTV more than 200 cm^3 ^belong to a relatively advanced cancer stage that is beyond the University of California at San Francisco criteria for liver transplantation [28,29]. Although selecting a cutoff to define the mortality risk may be arbitrary in patients undergoing different therapeutic strategies, these two cutoffs of TTV may be used for future reference in prognostic prediction.

The role and clinical significance of using TTV as a prognostic predictor were validated in the staging system for HCC. The CLIP scoring system is a widely used model for prognostic stratification [8,30]. We found that using TTV in the CLIP system could accurately predict the outcome of patients in different score groups (Figure 3). This result suggests that TTV is a feasible alternative indicator of tumor burden for cancer staging.

An important implication of this study is that patients with multi-nodular lesions may not necessarily have a larger TTV (Table 2). This is because many patients had a main tumor and several satellite lesions which could be quite small. As a result, the calculated TTV in patients with more tumor nodules was not necessarily larger than that of patients with fewer number of tumor nodules. This finding implicates that by reporting the size and number of tumor nodule in HCC patients may not be sufficient and sometimes could be misleading if the actual tumor size for individual tumor nodule is not mentioned. In this regard, reporting the value of TTV instead of the size and number of tumor nodule may more accurately indicate the extent of tumor involvement in liver.

TTV was also closely linked with serum AFP level and the presence of vascular invasion. Both of these two factors are important tumor-associated parameters and were also independent prognostic predictors in our analysis. Abundant studies consistently showed that these two predictors may predict the survival of HCC patients undergoing either surgical or non-surgical therapy [31-39]. Since TTV can be used to reflect the status of tumor burden, it is not surprising that a larger TTV is expected to be more often associated with a higher AFP level and vascular invasion. By contrast, it is anticipated that TTV is not related to the severity of liver cirrhosis, as indicated by the MELD and CTP score, in this study. These findings suggest that TTV is an authentic tumoral factor independent of the severity of liver cirrhosis.

In addition to serum AFP level, vascular invasion and TTV, a higher CTP score and performance status were also identified as independent prognostic predictors in this study. A higher CTP score is known to correlate with the severity of liver cirrhosis and could independently predict the mortality risk. Performance status represents the overall physical reserve in a given patient. This parameter has been exclusively included in the BCLC system which was suggested as the primary staging system for HCC [40], and our findings further confirm its role for prognostic prediction.

There are nevertheless a few potential limitations of this study. Firstly, a shortcoming of using TTV is that the estimation of tumor volume is based on the assumption that all tumors are spherical. This estimate might be erroneous if the tumor is irregular shaped. Secondly, our results were derived from patients undergoing locoregional therapy, therefore the strategy for selecting appropriate cutoffs of TTV for patients receiving other treatment modalities may not be the same. Thirdly, the treatments used in individual patient were heterogeneous depending on the status of tumor spread and severity of cirrhosis. This may interfere with the assessment of the impact of TTV on survival. Lastly, the TTV in HCC patients with extrahepatic metastasis was not considered in this study. The role of TTV as a prognostic predictor in these far-advanced cancer stage patients needs further studies to clarify.

## Conclusions

Our results indicate that TTV is a feasible prognostic predictor across a wide range of gradient and can be used to predict the mortality risk in HCC patients undergoing locoregional therapy. TTV can be easily and readily obtained, and future clinical trials of HCC may include this parameter for mortality risk stratification. Selecting appropriate cutoffs of TTV may help refine the design of cancer staging system and fine-tuning of treatment planning.

## Abbreviations

AFP: α-fetoprotein; (AIC): Akaike information criterion; ALT: alanine aminotransferase; AST: aspartate aminotransferase; CI: confidence interval; CLIP: Cancer of the Liver Italian Program; CTP: Child-Turcotte-Pugh; ECOG: Eastern Cooperative Oncology Group; HBV: hepatitis B virus; HCV: hepatitis C virus; HCC: hepatocellular carcinoma; INR: international normalized ratio; MELD: model for end-stage liver disease; PAI: percutaneous acetic acid injection; PEI: percutaneous ethanol injection; RFA: radiofrequency ablation; RR: relative risk; SD: standard deviation; TACE: transarterial chemoembolization; TTV: total tumor volume

## Competing interests

The authors declare that they have no competing interests.

## Authors' contributions

Data acquisition and analysis, and manuscript preparation were performed by TIH, CYH and YHH. Patient enrollment, treatment and follow-up were performed by HCL, CWS, RCL, JHC and YYC. PCL was responsible for study design and statistical analysis. Critical manuscript review was performed by SDL. All authors have read and approved the final manuscript.

## Pre-publication history

The pre-publication history for this paper can be accessed here:

http://www.biomedcentral.com/1471-230X/10/146/prepub
